# Hexavalent Chromium Cr(VI) Up-Regulates COX-2 Expression through an NFκB/c-Jun/AP-1–Dependent Pathway

**DOI:** 10.1289/ehp.1104179

**Published:** 2012-01-06

**Authors:** Zhenghong Zuo, Tongjian Cai, Jingxia Li, Dongyun Zhang, Yonghui Yu, Chuanshu Huang

**Affiliations:** 1 Nelson Institute of Environmental Medicine, New York University School of Medicine, Tuxedo, New York, USA; 2 Key Laboratory of the Ministry of Education for Coast and Wetland Ecosystems, School of Life Sciences, Xiamen University, Xiamen, China

**Keywords:** AP-1, chromium, c-Jun, COX-2, NFκB

## Abstract

Background: Hexavalent chromium [Cr(VI)] is recognized as a human carcinogen via inhalation. However, the molecular mechanisms by which Cr(VI) causes cancers are not well understood.

Objectives: We evaluated cyclooxygenase-2 (COX-2) expression and the signaling pathway leading to this induction due to Cr(VI) exposure in cultured cells.

Methods: We used the luciferase reporter assay and Western blotting to determine COX-2 induction by Cr(VI). We used dominant negative mutant, genetic knockout, gene knockdown, and chromatin immunoprecipitation approaches to elucidate the signaling pathway leading to COX-2 induction.

Results: We found that Cr(VI) exposure induced COX-2 expression in both normal human bronchial epithelial cells and mouse embryonic fibroblasts in a concentration- and time-dependent manner. Deletion of IKKβ [inhibitor of transcription factor NFκB (IκB) kinase β; an upstream kinase responsible for nuclear factor κB (NFκB) activation] or overexpression of *TAM67* (a dominant-negative mutant of c-*Jun*) dramatically inhibited the COX-2 induction due to Cr(VI), suggesting that both NFκB and c-Jun/AP-1 pathways were required for Cr(VI)-induced COX-2 expression. Our results show that p65 and c-Jun are two major components involved in NFκB and AP-1 activation, respectively. Moreover, our studies suggest crosstalk between NFκB and c-Jun/AP-1 pathways in cellular response to Cr(VI) exposure for COX-2 induction.

Conclusion: We demonstrate for the first time that Cr(VI) is able to induce COX-2 expression via an NFκB/c-Jun/AP-1–dependent pathway. Our results provide novel insight into the molecular mechanisms linking Cr(VI) exposure to lung inflammation and carcinogenesis.

Chromium (Cr) is a ubiquitous metal found in animals, plants, rocks, soil, and air (Hill et al. 2008). Exposure to hexavalent chromium [Cr(VI)] occurs in multiple occupational environments, and the approximate daily absorbed dose of Cr(VI) is 83–1,700 μg/kg/day (Beveridge 2010). The International Agency for Research on Cancer (1980) has classified Cr(VI) as a known human carcinogen. Previous *in vivo* studies strongly indicated that there is an association between Cr(VI) exposure and airway inflammation and lung carcinogenesis (Beaver et al. 2009a, 2009b; Zeidler-Erdely et al. 2008). However, the molecular mechanisms by which Cr(VI) induces lung inflammation and cancers are not yet well understood.

Prostaglandin (PG) is an important mediator at all stages of cancer development (Menter 2002). Cyclooxygenase (COX) is the rate-limiting enzyme in the synthesis of PGs (Rao et al. 2004). The COX enzyme system is composed of two isoenzymes: COX-1, the constitutive isoform, and COX-2, the inducible protein (Davies et al. 2002). COX-2 can undergo rapid induction in response to many factors, such as growth factors and cytokines (Kirschenbaum et al. 2001), and is highly expressed in a variety of human cancers and cancer cell lines (Liao and Milas 2004). COX-2 overexpression is associated with more aggressive biological tumor behaviors (Liao and Milas 2004), and the inhibition of COX-2 has been regarded as an effective anticancer strategy (Davies et al. 2002). Thus, identification of the potential involvement of COX-2 and molecular mechanisms responsible for COX-2 induction due to Cr(VI) exposure will provide significant insight into understanding Cr(VI) lung inflammatory and carcinogenic effects. In the present study, we investigated the potential effects of Cr(VI) on COX-2 expression and molecular mechanisms leading to this induction in cell culture models.

## Materials and Methods

*Cell culture and reagents.* Mouse embryonic fibroblasts (MEFs) were cultured in 37°C with 5% CO_2_ in Dulbecco’s modified Eagle’s medium (DMEM) supplemented with 10% fetal bovine serum (FBS),1% penicillin/streptomycin, and 2 mM l-glutamine (all from Life Technologies, Grand Island, NY, USA) (Song et al. 2008). Normal human bronchial epithelial cells (NHBECs) were cultured in a modified LHC-9 medium (BioWhittaker, Inc., Walkersville, MD, USA) supplemented with 52 μg/mL bovine pituitary extract, 0.5 μg/mL hydrocortisone, 0.5 ng/mL human epidermal growth factor, 0.5 μg/mL epinephrine, 10 μg/mL transferrin, 5 μg/mL insulin, 0.1 ng/mL retinoic acid, 6.5 ng/mL triiodothyronine, 50 μg/mL gentamicin, and 50 ng/mL amphotericin-B. We purchased antibodies specific for IκBα [inhibitor of transcription factor NFκB-α), phosphorylated IκBα (P-IκBα), c-Jun, phosphorylated c-Jun (P-c-Jun73), and IκB kinase β (IKKβ) from Cell Signaling Technology (Beverly, MA, USA); Antibodies against COX-2, Jun-B, Jun-D, c-Fos, Fra-1, and p65 from Santa Cruz Biotechnology (Santa Cruz, CA, USA); anti-β-actin antibody from Sigma (St. Louis, MO, USA) and Sungene Biotech (Tianjin, China); and anti-p50 antibody from Abcam (Cambridge, MA, USA). The luciferase assay substrate was purchased from Promega (Madison, WI, USA), and sodium chromate (Na_2_CrO_4_) was purchased from Aldrich (Milwaukee, WI, USA).

*Plasmid constructs and transfection.* We purchased AP-1–luciferase (AP-1-Luc) plasmid from Stratagene (Santa Clara, CA, USA). The COX-2-Luc reporter plasmid, nuclear factor of activated T cells (NFAT)-Luc reporter plasmid, nuclear factor κB (NFκB)-Luc reporter plasmid, dominant-negative mutant of *IKK*β (IKKβ-KM), hemagglutinin-tagged IKKβ (HA-IKKβ), and c-Jun dominant-negative mutant (pcDNA3.1/His-TAM67), as well as IKKβ^−/−^ and IKKα^−/−^ MEFs and their corresponding wild-type (WT) MEFs, were described previously (Ding et al. 2006b; Ouyang et al. 2007a; Tang et al. 2001). Transfection experiments were performed with Lipofectamine 2000 reagent (Invitrogen, Carlsbad, CA, USA) following the manufacturer’s instructions. The stable transfectants were established and cultured in antibiotic-free DMEM for at least two passages before performing experiments.

*Reverse-transcription polymerase chain reaction (RT-PCR).* After the cells were treated with Na_2_CrO_4_, total RNA was extracted using TRIZOL reagent (Invitrogen) following the manufacturer’s instructions. First-strand cDNA was synthesized with oligo(dT)_20_ primers using the SuperScript III First-Strand Synthesis System for RT-PCR; Invitrogen), and 1 μg of total RNA was used to perform reverse transcription. Specific primer pairs were designed for amplifying murine *cox-2* (forward, 5´-tca ccc gag gac tcc gcc-3´; reverse, 5´-tcc tgc ccc aca gca aac tgc-3´) and β-actin (forward, 5´-gac gat gat att gcc gca ct-3´; reverse, 5´-gat acc acg ctt gct ctg ag-3´). For specific amplifications, 50 ng of cDNA templates was used.

*Luciferase reporter assay.* MEFs transfected with the luciferase reporter constructs were seeded into 96-well plates (8 × 10^3^/well) and subjected to various treatments when cultures reached 80–90% confluence. For ultraviolet B (UVB) radiation, culture plates were covered with a thin layer of fresh medium (0.1% FBS-DMEM) and exposed to UVB light for 1 min, corresponding to a dose of 1 kJ/m^2^, as reported previously (Song et al. 2007). The UVB light source (UVP Inc., Upland, CA, USA) emitted > 95% 302-nm UVB light. Luciferase activity was determined using a luminometer (Wallac 1420 Victor 2 multilabel counter system; PerkinElmer, Waltham, MA, USA) as described previously (Huang et al. 2002). The results are expressed as relative activity normalized to the luciferase activity in the control cells without treatment.

*Western blotting assay.* Cells (2 × 10^5^) were seeded and cultured in each well of six-well plates until 70–80% confluence. The cells were exposed to Cr(VI) at varying doses and time points and then extracted with sodium dodecyl sulfate sample buffer as previously described (Ouyang et al. 2007b). The cell extracts were used for Western blotting with specific antibodies. The protein band, specifically bound to the primary antibody, was detected using an anti-rabbit IgG-alkaline phosphatase (AP)–linked antibody and an electrochemifluorescence (ECF) Western blotting system (Amersham Biosciences, Piscataway, NJ, USA). The images were obtained by scanning using the Storm 860 phosphoimager (Molecular Dynamics, Sunnyvale, CA, USA)

*Electrophoretic mobility shift assay (EMSA) and super gel shift.* We performed the EMSA using the LightShift Chemiluminescent EMSA Kit (Pierce, Rockford, IL, USA) according to the manufacturer’s instructions. Nuclear extracts were isolated with a Nuclear/Cytosol Fractionation Kit (BioVision, Mountain View, CA, USA). The specific probe pair designed for activated NFκB was 5´-agt tga ggg gac ttt ccc agg c-3´ and 5´-gcc tgg gaa agt ccc ctc aac t-3´. The specific probe pair designed for activated AP-1 was 5´-cgc ttg atg agt cag ccg gaa-3´ and 5´-ttc cgg ctg act cat caa gcg-3´. The probes were conjugated with biotin by a Biotin 3´ End DNA Labeling Kit (Pierce) following the manufacturer’s instructions. Nuclear protein (4 µg) was subjected to the gel shift assay by incubation with 1 μg poly(dI-dC) DNA carrier in DNA binding buffer [10 mM Tris (pH 8.0), 150 mM potassium chloride, 2 mM EDTA, 10 mM magnesium chloride, 10 mM dithiothreitol, 0.1% bovine serum albumin, 20% glycerol]. The biotin-labeled double-stranded oligonucleotide (1 μL) was then added, and the reaction mixture was incubated at room temperature for 50 min. For competition experiments, a 50-fold molar excess of the unlabeled double-stranded oligonucleotide was added before the addition of the labeled probe. For the super gel shift assay, nuclear extracts were incubated with 2 μg antibody for 30 min at 4°C before addition of the probe. DNA–protein complexes were resolved by electrophoresis on 5% nondenaturing glycerol-polyacrylamide gels. The luminescent signal was developed by a LightShift® Chemiluminescent EMSA Kit and detected by an automatic developing machine.

*Chromatin immunoprecipitation (ChIP) assay.* The ChIP assay was performed using the EZ ChIP kit (Upstate, Billerica, MA, USA) according to the manufacturer’s instructions. Briefly, cells were either untreated or treated with Cr (20 μM) for 12 hr, and then genomic DNA and the proteins were cross-linked with 1% formaldehyde. The cross-linked cells were pelleted, resuspended in lysis buffer, and sonicated to generate 200- to 500-bp chromatin DNA fragments. After centrifugation, the supernatants were diluted 10-fold and then incubated with anti-p65 or anti-c-Jun antibodies, respectively, or the control rabbit IgG at 4°C overnight. The immune complex was captured by protein G agarose saturated with salmon sperm DNA and then eluted with elution buffer. DNA–protein cross-linking was reversed by heating at 65°C for 4 hr. DNA was purified and subjected to PCR analysis.

To specifically amplify the region containing the putative NFκB-responsive elements on the mouse *COX-2* promoter, we performed PCR using the following primers: 5´-ctg acg agc gag cac gtc-3´ (forward) and 5´-ttt ggc ctc tgg ggt ttc-3´ (reverse). To specifically amplify the region containing the putative AP-1–responsive elements on the mouse *COX-2* promoter, PCR was performed with the following primers: 5´-ttc cca taa gac tcc g-3´ (forward) and 5´-gct tca tgt gca agc t-3´ (reverse). Primers targeting the region 1 kb upstream of the NFκB and AP-1 binding sites on the *COX-2* promoter were also used in the PCR analysis to support the specificity of the ChIP assay: 5´-tga ttt ggt ttg gga ca-3´ (forward) and 5´-ctg gag gac aag agc agt-3´ (reverse).

*Clonogenic survival assay.* MEFs were treated with Cr(VI) at 5 μM and 20 μM for 6 and 12 hr and recovered for 24 hr in normal culture medium. Cells were then plated at 500 cells/dish in 100-mm cell culture dishes and cultured for 2 weeks. Cells were stained with Giemsa solution, and the number of colonies was counted and presented as mean ± SD (*n* = 3).

*Statistical analysis.* We used the Student’s *t*-test to determine the significance of difference in COX-2 induction and AP-1, NFAT, or NFκB activation in luciferase reporter assays among various groups. The statistical significance level was set at *p* < 0.05.

## Results

**Figure 1:**
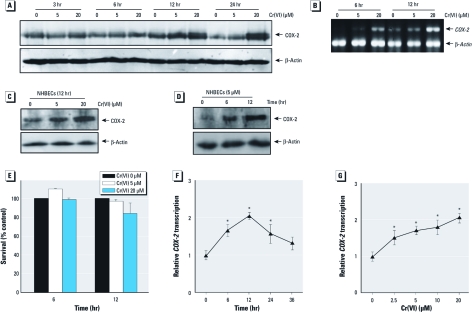
Cr(VI) exposure resulted in COX-2 induction. WT MEFs (*A,B*) or NHBECs (*C,D*) were exposed to Cr(VI) as indicated. The cells were extracted and COX-2 expression was determined by Western blotting (*A*,*C,D*) or by RT-PCR (*B*). β‑Actin was used as a loading control. (*E*) WT MEFs were treated with Cr(VI) at indicated doses for 6 and 12 hr and then allowed to recover in normal culture medium for 24 hr; cytotoxicity was determined by colony survival assay. (*F,G*) COX-2 promoter–driven luciferase transcription relative to control (relative *COX-2 *transcription) was determined in MEFs treated with 20 μM Cr(VI) for various times (*F*) or at different Cr(VI) doses for 12 hr (*F*). Data are mean ± SD of triplicates. *p < 0.05, compared with control cells (medium only).

*Cr(VI) exposure induced COX-2 expression.* As shown in Figure 1A, treatment of MEFs with Cr(VI) resulted in an increase in COX-2 protein expression in a dose- and time-dependent manner. We observed marked induction at 12 hr and 24 hr after exposure. Cr(VI) exposure was previously reported to induce either cell growth arrest and/or apoptosis in a dose-, time- and, cell-type–dependent manner (Wang et al. 2004). To evaluate the cytotoxicity of Cr(VI) in our experimental system, we subjected Cr(VI)-treated MEFs to a colony-survival assay. Results showed only marginal toxicity on MEFs exposed to 20 μM Cr(VI) after 12 hr of exposure, whereas there was no observable cytotoxicity at 5 μM (Figure 1E). These results are consisted with a previous report showing that the viability of HaCaT (human keratinocyte) cells is not affected at Cr(VI) concentrations as high as 30 μM (Wang et al. 2010). Consistent with protein induction, marked induction of *COX-2* mRNA by 20 μM Cr(VI) was present as early as 6 hr after exposure, suggesting that Cr(VI) might induce *COX-2* expression at a transcriptional level (Figure 1B). To test this notion, we investigated the effects of Cr(VI) on *COX-2* promoter activity in the stable transfectant of *COX-2* promoter–driven luciferase reporter. As shown in Figure 1F and 1G, treatment with Cr(VI) resulted in a marked increase in *COX-2* promoter activity. This induction was also observed with 20 μM Cr(VI) as early as at 6 hr after exposure (Figure 1F), which is consistent with the results of the RT-PCR assay. The respiratory tract is the primary target organ of Cr(VI) (Goldoni et al. 2008). Thus, we used NHBECs to test the effect of Cr(VI) on COX-2 expression. Cr(VI) exposure did cause COX-2 expression in NHBECs (Figure 1C,D). Collectively, these results indicate that Cr(VI) is able to induce COX-2 expression in both MEFs and NHBECs.

**Figure 2:**
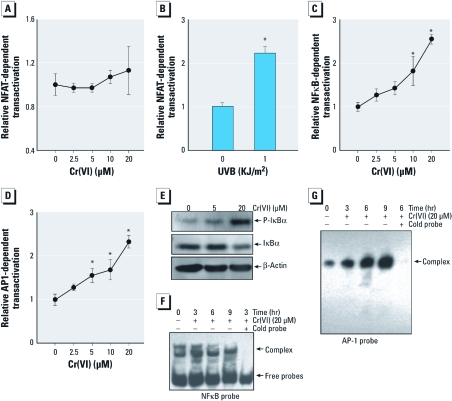
Cr(VI) treatment induced the activation of NFκB and AP-1 but not NFAT. (*A–D*) NFAT-dependent (*A* and *B*), NFκB-dependent (*C*), and AP-1–dependent (*D*) transactivation in MEFs was determined by specific luciferase reporter assay after exposure to different concentrations of Cr(VI) for 12 hr or UVB (1 kJ/m^2^) for 6 hr. Values are mean ± SD of triplicates. (*E*) MEFs were exposed to Cr(VI) for 1 hr and then extracted and subjected to Western blotting analysis. β‑Actin was used as a loading control. (*F,G*) MEFs were exposed to 20 μM Cr(VI), and the nuclear extracts were subjected to the gel shift assay with NFκB (*F*) or AP-1 (*G*) probe. For competition experiments, a 50‑fold molar excess of unlabeled NFκB or AP-1 cold probe was added to the binding reaction mixtures to determine the specific binding. *p < 0.05, compared with control.

*Cr(VI) exposure induced the activation of NF*κ*B and AP-1 but not NFAT.* Cr(VI) treatment did not result in observable NFAT activation (Figure 2A), whereas UVB exposure, the positive control, resulted in significant NFAT activation (Figure 2B) in the same stable NFAT-Luc reporter transfectant. In contrast to NFAT, NFκB activation was significantly increased by Cr(VI) treatment in the NFκB-Luc reporter assay (Figure 2C). The activation of the NFκB pathway by Cr(VI) was further verified by the observation of increased IκBα phosphorylation and degradation in the Western blotting assay (Figure 2E) and NFκB DNA binding activity analyzed by an EMSA assay (Figure 2F). We further determined the involvement of the AP-1 pathway in cells exposed to Cr(VI). As shown in Figure 2D and 2G, treatment of cells with Cr(VI) for 6 hr also led to marked AP-1 induction in the AP-1-Luc reporter assay (Figure 2D) and the AP-1 EMSA assay (Figure 2G). These results demonstrate that Cr(VI) exposure induced activation of NFκB and AP-1 but not NFAT.

DNA binding activity of NFκB induced by Cr(VI) reached to peak at 3 hr (Figure 2F), whereas the maximum *AP-1* DNA binding activity was achieved at 9 hr after exposure (Figure 2G). The difference could be due to the differential pathways responsible for activation of NFκB and AP-1. NFκB activation is fully dependent on IKKβ/IκB phosphorylation/degradation (Song et al. 2006), whereas AP-1 activation is dependent on both c-Jun phosphorylation and increased c-Jun protein expression (Huang et al. 1999a, 1999b). The induction of c-Jun protein expression may lead to the delay of maximum AP-1 activation compared with the peak of NFκB activation. Cr(VI) has been reported to inhibit tumor necrosis factor-α–induced NFκB transcriptional competence through inhibiting interactions with coactivators of transcription rather than DNA binding (Shumilla et al. 1999). Another study found that Cr(VI) prevented the benzo[*a*]pyrene-dependent release of histone deacetylase-1 from cytochrome P450 1a1 chromatin and blocked p300 recruitment (Wei et al. 2004).

**Figure 3:**
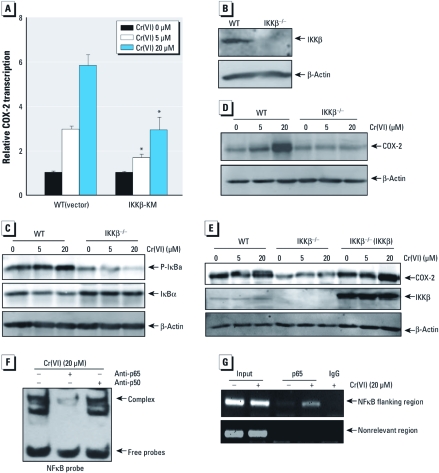
IKKβ/NFκB activation is required for Cr(VI)-induced COX-2 expression in MEFs. (*A*) MEFs were exposed to Cr(VI) for 6 hr, and the luciferase activities were determined; results are expressed as COX-2 induction relative to control. (*B*) IKKβ expression in WT and IKKβ^–/–^ MEFs. (*C*–*E*) WT (vector), IKKβ^–/–^ (vector), and IKKβ^–/–^ (IKKβ) MEFs were seeded into six-well plates, and Western blotting analysis was performed with anti–P‑IκBα and anti‑IκBα (*C*), anti‑COX‑2 (*D*), or anti‑COX‑2 and anti‑IKKβ (*E*). β‑Actin was used as a loading control. (*F*) MEFs were exposed to 20 μM Cr(VI) for 3 hr, and then the nuclear extracts were subjected to a super gel shift assay using anti‑p65 and anti‑p50. (*G*) MEFs were exposed to 20 μM Cr(VI) for 3 hr, and then the ChIP assay was performed. *p < 0.05, compared with WT (vector) cells.

*IKK*β *is required for CI(VI)-induced COX-2 expression.* To clarify the potential role of IKKβ in Cr(VI)-induced COX-2 expression, we used IKKβ-KM, an inactive mutant of IKKβ, and IKKβ^−/−^ MEFs. As shown in Figure 3A, overexpression of IKKβ-KM in MEFs inhibited Cr(VI)-induced COX-2 expression in the COX-2-Luc reporter assay. The knockout of IKKβ (Figure 3B) impaired the phosphorylation and degradation of its downstream target IκBα after Cr(VI) treatment (Figure 3C), indicating the necessary role of IKKβ in Cr(VI)-induced NFκB activation. Cr(VI)-induced COX-2 protein expression was consistently blocked in IKKβ^−/−^ cells (Figure 3D). Moreover, reconstituted expression of IKKβ in IKKβ^−/−^ cells restored COX-2 induction (Figure 3E). Our results demonstrate that IKKβ was required for COX-2 induction after Cr(VI) exposure. Overexpression of IKKβ-KM was not able to completely inhibit *COX-2* promoter–driven luciferase transcription (Figure 3A), whereas IKKβ deletion (IKKβ^−/−^) was able to block COX-2 expression completely (Figure 3D). These results suggest that IKKβ-KM overexpression was not able to completely impair endogenous IKKβ function.

*The potential role of NF*κ*B p65 in the regulation of COX-2 expression due to Cr(VI) exposure.* NFκB components are expressed in a variety of cell types (Karin and Greten 2005). In a previous study we showed that the NFκB p65 subunit, but not the p50 subunit, is required for nickel-induced COX-2 expression in Beas-2B cells (Ding et al. 2006b). In the present study, we determined the differential involvement of p65 and p50 subunits in Cr(VI)-induced COX-2 expression. We performed a super gel shift assay in the presence of the antibodies specific for p65 or p50. As shown in Figure 3F, selective reduction of the p65 band was observed using anti-p65 antibody, whereas no reduction of DNA binding activity was observed with anti-p50 antibody. Incubation of cell nucleus extracts with anti-p65 antibody reduced the extract protein binding to the NFκB probe but did not cause the supershift band. The explanation for this may be that binding of anti-p65 antibody to p65 protein changes the p65 protein conformation and in turn leads to p65 losing its binding activity to the NFκB probe. These results suggest that p65 might be the major component involved in NFκB activation after Cr(VI) exposure. This notion is further supported by ChIP assay data. As shown in Figure 3G, Cr(VI) treatment markedly enhanced recruitment of the p65 subunit to its binding site in *COX-2* promoters, whereas control IgG and primers targeting the DNA sequence located at approximately 1 kb upstream of the NFκB binding site in the *COX-2* promoter did not show detectable PCR products (Figure 3G). Taken together, these results demonstrate that NFκB p65, rather than the p50 subunit, plays a key role in NFκB activation and COX-2 induction after Cr(VI) exposure.

**Figure 4:**
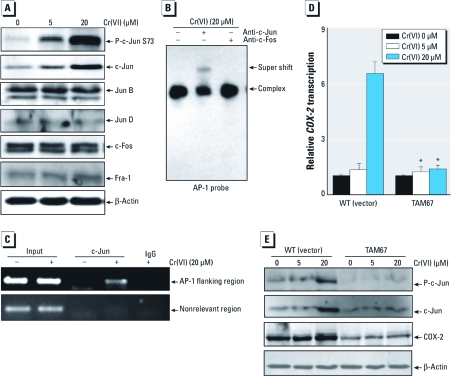
Requirement of c-Jun/AP-1 for Cr(VI)-induced COX-2 expression. (*A*) MEF cells were exposed to Cr(VI) for 12 hr, and cell extracts were subjected to Western blotting. (*B*) MEFs were exposed to 20 μM Cr(VI) for 6 hr, and nuclear extracts were subjected to a super gel shift assay for c‑Jun and c‑Fos. (*C*) MEFs were exposed to 20 μM Cr(VI) for 3 hr, before performing the ChIP assay. (*D*) MEFs transiently transfected with the COX-2‑Luc reporter construct or COX-2‑Luc reporter together with a c-*Jun* mutant construct (TAM67) were then exposed to Cr(VI), and the luciferase activities were determined 6 hr after treatment. Results are expressed as COX-2 induction relative to control. (*E*) WT (vector) or TAM67 MEFs cells were treated with Cr(VI) for 24 hr, and cell extracts were subjected to Western blotting. β‑Actin was used as a loading control. *p < 0.05, compared with WT (vector) MEFs.

*Involvement of c-Jun/AP-1 in Cr(VI)-induced COX-2 expression.* Different AP-1 dimers play different roles in the regulation of cellular function and carcinogenesis (Song et al. 2008). Western blotting shows that Cr(VI) exposure resulted in c-Jun phosphorylation, but we observed no activation of other AP-1 members Jun B, Jun D, c-Fos, or Fra-1 (Figure 4A). To determine the role of c-Jun in Cr(VI)-induced AP-1 activation, we performed a super gel shift assay using antibodies specific for c-Jun and c-Fos. As shown in Figure 4B, we observed a selective supershift band of c-Jun in cell extracts from Cr(VI)-treated cells, but no c-Fos supershift band was observable, suggesting that c-Jun was the major component involved in AP-1 activation due to Cr(VI) exposure. *COX-2* has been shown to be a typical AP-1–regulated gene in several experimental systems (Zhang et al. 2010). Thus, we determined the recruitment of c-Jun to the *COX-2* promoter region using the ChIP assay. The detection of the *COX-2* promoter in the antibody-captured genomic DNA fragments was performed by PCR amplification with primers designed to specifically recognize the region containing AP-1–responsive elements. Anti-c-Jun antibody strongly coimmunoprecipitated the target *COX-2* promoter region DNA in Cr(VI)-treated cell extract but not in the control cell extract (Figure 4C), indicating the inducible recruitment of c-Jun to the endogenous *COX-2* promoter after Cr(VI) exposure. This demonstrates Cr(VI)-inducible recruitment of AP-1 onto the endogenous *COX-2* promoter region (Figure 4C), suggesting that AP-1 might play a role in the regulation of COX-2 expression due to Cr(VI) exposure. To test this notion, we used *TAM67*, a dominant negative mutant of c-*Jun*. The ectopic expression of TAM67 in WT cells attenuated Cr(VI)-induced c-Jun phosphorylation in MEFs (Figure 4E). Unlike overexpression of IKKβ-KM in MEFs (Figure 3A), *COX-2* promoter–driven luciferase transcription was impaired in WT/TAM67 transfectant (Figure 4D), suggesting that TAM67 overexpression was able to block the endogenous c-Jun function. COX-2 protein induction by Cr(VI) was also blocked (Figure 4E). These results demonstrate that c-Jun activation is essential for COX-2 induction after Cr(VI) exposure.

**Figure 5:**
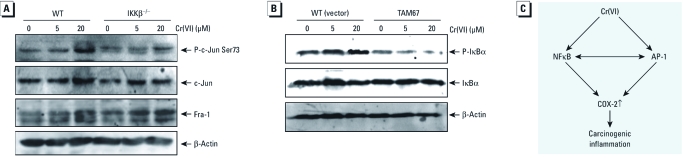
Crosstalk between AP-1 and the NFκB pathway after Cr(VI) exposure. (*A* and *B*) WT and IKKβ^–/–^ (*A*) or WT (vector) and TAM67 (*B*) MEFs were treated with Cr(VI) for 1 hr, and cell extracts were subjected to Western blotting. β‑Actin was used as a loading control. (*C*) The overall scheme for Cr(VI)-induced COX-2 expression.

*Crosstalk between AP-1 and NF*κ*B pathways after Cr(VI) exposure.* Crosstalk between AP-1 and NFκB has been reported to be responsible for the synergistic increase in their activity in the regulation of target gene expression (Adcock 1997). Thus, we determined the potential relationship of these two transcription factors in response to Cr(VI) exposure in cells. We used IKKβ^−/−^ MEFs to examine whether the impairment of the NFκB pathway could affect c-Jun phosphorylation. Impairment of the NFκB pathway inhibited c-Jun phosphorylation (Figure 5A), suggesting that NFκB activation has a positive effect on c-Jun activation after Cr(VI) exposure. To further reveal the potential effects of c-Jun/AP-1 on NFκB activation, we used a dominant negative mutant of c-Jun (TAM67). As shown in Figure 5B, ectopic expression of TAM67 had an inhibitory effect on IκBα phosphorylation, suggesting that the phosphorylation of c-Jun was also involved in the regulation of the NFκB pathway. Taken together, the AP-1 and NFκB pathways did show crosstalk after Cr(VI) treatment, which might play a role in Cr(VI)-induced COX-2 induction and carcinogenesis (Figure 5C).

## Discussion

The data we present here indicate that Cr(VI) induced expression of COX-2 and activation of AP-1 and NFκB, and show that both AP-1 and NFκB are required for Cr(VI)-induced COX-2 expression. Our data also indicate the presence of crosstalk between the NFκB and AP-1 pathways after Cr(VI) exposure, which mainly occurred via IKKβ/p65-dependent and c-Jun–dependent pathways. Considering the important role of COX-2 in the mediation of chronic inflammation and lung carcinogenesis, we anticipated that activation of NFκB and AP-1 pathways and their crosstalk in the regulation of COX-2 expression might be key factors in Cr(VI)-induced lung carcinogenesis. Further elucidating the relationship among chronic inflammation, COX-2 induction, and lung carcinogenic effect after various doses of Cr(VI) exposure *in vivo* animal models will be a major focus for future investigations in our laboratory, which might help determine a threshold dose for lung carcinogenesis of Cr(VI) exposure.

Inflammation is implicated in Cr(VI)-induced human lung cancer development. Repetitive exposure to Cr(VI) results in persistent inflammation, and such an inflammatory microenvironment can further promote lung carcinogenesis (Beaver et al. 2009a, 2009b). COX-2 plays an important role in the development of various types of cancer, including lung cancer (Sahin et al. 2009), and drugs targeting this enzyme have achieved widespread clinical use (Bertagnolli 2007). Our previous studies have shown that COX-2 induction is involved in several carcinogenic responses (Ding et al. 2006a, 2006b; Li et al. 2006). In the present study, we initially found that exposure to Cr(VI) induced COX-2 expression in both NHBECs and MEFs. Considering the critical role of COX-2 in the inflammatory processes of cancer and the importance of an inflammatory microenvironment during carcinogenesis after Cr(VI) exposure, our results may shed light into the mechanisms of Cr(VI)-induced carcinogenic effects.

The *COX-2* promoter region contains the binding sites of three major transcription factors: NFκB (Crofford et al. 1997), AP-1 (Subbaramaiah et al. 2002), and NFAT (Iniguez et al. 2000). These three factors have been reported to be major mediators for the regulation of cell proliferation, differentiation, and transformation (Huang et al. 1999a, 1999b). In the present study, we observed that Cr(VI) exposure resulted in the activation of NFκB and AP-1, whereas there was no observable NFAT activation, which is consistent with published studies showing that Cr(VI) exposure leads to the activation of NFκB and AP-1 in an oxidative-stress–dependent manner (Yao et al. 2008). NFκB activation has been reported to be involved in the development of several cancers (Biswas et al. 2004; Wang et al. 2003). Our published studies have shown that NFκB activation is involved in cellular responses to several environmental carcinogens (Ding et al. 2007; Ouyang et al. 2007b). In the present study, we found that IKKβ was critical for Cr(VI)-induced NFκB activation and COX-2 expression. In addition, we showed that p65, rather than p50, was required for Cr(VI)-induced NFκB activation and COX-2 expression. We observed that Cr(VI) exposure induces NFκB activation via an IKKβ/p65-dependent pathway, which further leads to COX-2 induction. Cr(VI) increases formation of reactive oxygen species (ROS) in certain cell types (Wang et al. 2010), and the inductive COX-2 expression of manganese is accompanied by generation of oxidative stress and increased NFκB and AP-1 DNA binding activities (Chen et al. 2007). Thus, we anticipate that ROS generation may also be involved in the activation of NFκB and AP-1, which further leads to COX-2 expression.

The c-Jun/AP-1 pathway is crucial for COX-2 induction caused by some environmental stresses (Ouyang et al. 2007a; Zhang et al. 2008). Because of the multiple functions of AP-1 proteins, the selection of the different AP-1 dimers is considered as another mechanism for the modulation of AP-1 activity (Song et al. 2008). The results of the present study indicate that AP-1 activation due to Cr(VI) exposure mainly involves c-Jun phosphorylation. The predominant role of c-Jun in Cr(VI)-induced AP-1 transactivation and COX-2 induction was further confirmed by super gel shift assay and ChIP assay. Furthermore, transfection with the dominant negative c-*Jun* mutant (TAM67) blocked Cr(VI)-induced COX-2 expression. In addition, the knockout of IKKβ impaired Cr(VI)-induced c-Jun phosphorylation, whereas inhibition of the c-Jun/AP-1 pathway by overexpression of TAM67 also inhibited Cr(VI)-induced IκBα phosphorylation, suggesting crosstalk between the c-Jun/AP-1 pathway and the IKKβ/NFκB pathway in the Cr(VI) response. Because both the c-Jun/AP-1 pathway and the IKKβ/NFκB pathway are crucial for COX-2 induction, we anticipate that this crosstalk may play a key role in Cr(VI)-induced COX-2 expression, which provides a novel model of the interaction between NFκB and AP-1 pathways for environmental responses. Considering that inhibition of NFκB, AP-1, and COX-2 has been proposed as potential anticancer strategies, our results may lead to new targets for chemoprevention of Cr(VI)-induced human carcinogenesis.
